# Rapid Life-History Diversification of an Introduced Fish Species across a Localized Thermal Gradient

**DOI:** 10.1371/journal.pone.0088033

**Published:** 2014-02-04

**Authors:** Fengyue Zhu, Andrew L. Rypel, Brian R. Murphy, Zhongjie Li, Tanglin Zhang, Jing Yuan, Zhiqiang Guo, Jianfeng Tang, Jiashou Liu

**Affiliations:** 1 State Key Laboratory of Freshwater Ecology and Biotechnology, Institute of Hydrobiology, the Chinese Academy of Sciences, Wuhan, China; 2 University of the Chinese Academy of Sciences, Beijing, China; 3 University of Wisconsin, Center for Limnology and Wisconsin Department of Natural Resources, Madison Wisconsin, United States of America; 4 Department of Fish and Wildlife Conservation, Virginia Polytechnic Institute and State University, Blacksburg Virginia, United States of America; Clemson University, United States of America

## Abstract

Climatic variations are known to engender life-history diversification of species and populations at large spatial scales. However, the extent to which microgeographic variations in climate (e.g., those occurring within a single large ecosystem) can also drive life-history divergence is generally poorly documented. We exploited a spatial gradient in water temperatures at three sites across a large montane lake in southwest China (Lake Erhai) to examine the extent to which life histories of a short-lived fish species (icefish, *Neosalanx taihuensis*) diversified in response to thermal regime following introduction 25 y prior. In general, warmwater icefish variants grew faster, had larger adult body size and higher condition and fecundity, but matured at smaller sizes. Conversely, coldwater variants had smaller adult body size and lower condition, but matured at larger sizes and had larger eggs. These life-history differences strongly suggest that key ecological trade-offs exist for icefish populations exposed to different thermal regimes, and these trade-offs have driven relatively rapid diversification in the life histories of icefish within Lake Erhai. Results are surprisingly concordant with current knowledge on life-history evolution at macroecological scales, and suggest that improved conservation management might be possible by focusing on patterns operating at microgeographical, including, within-ecosystem scales.

## Introduction

Biologists have long been fascinated with how thermal habitat variations shape species evolution and life histories [1]–[4]. For example, evolutionary biology is intensely focused on how climatic conditions drive the selection of genotypes leading to species formation [5]. Similarly, ecologists have explored diverse ecogeographic and macroecological hypotheses on how temperature affects populations and ecosystems by producing life-history variants [6]–[8]. For example, Bergmann's rule (i.e., that intraspecific variation in body size is negatively related to temperature) is one of the oldest and more-controversial topics in ecology and was originally theorized to result directly from the relationship between heat conservation and available surface area of animals [1]. The macroecology literature is in fact replete with examples of how large-scale variations in temperature and climate (e.g., as occurs across a continent) drive the development and maintenance of species and populations [9]–[13]. Considerably less is known on how microgeographic or meso-scale variations in temperature encourage life-history diversification, even though climate often varies dramatically at these spatial scales as well [14], [15].

One of the best opportunities for expanding this work lies in the simple observation that temperature often varies widely within a single large ecosystem [16]–[18]. Such within-system variations in thermal habitat can be significant enough that sub-populations derive genetic and phenotypic distinctiveness to varying degrees [19]–[21]. Understanding the spatial scale at which eco-thermal rules operate is now critical to the conservation management of diverse species and populations. In addition to multiple additive environmental threats from humans [22], climate change threatens to fundamentally unravel the thermal-habitats upon which species and life histories are adapted, thereby creating mal-adaptation and extinction risks [23]–[25].

**Figure 1:**
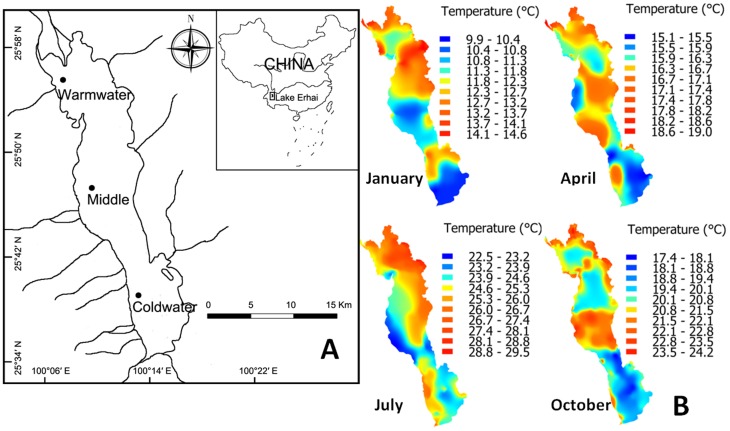
Sampling sites and heat map of Lake Erhai. (A) Location of icefish sampling sites in three discrete sections of Lake Erhai and (B) heat map for four months of the year documenting observed average water temperature variations across Lake Erhai.

Lake Erhai is a large alpine lake located in Yunnan province (southwestern China). Standing at the foot of the Cangshan Mountains, it has a surface area of 249.0 km^2^, is 42.6 km long from south to north, and has an average and maximum depth of 10.2 m and 20.7 m respectively. The lake receives inflow from two large rivers in the north and several smaller streams in the southwest. Previous limnological surveys revealed that water temperatures in the northern sections of the lake are consistently higher than temperatures in the southern sections with a typical difference of ∼1°C or more (Fig. 1). This temperature differential is generated by water derived from snowmelt and precipitation in the Cangshan Mountains flowing into the southern portions of the lake.

The icefish *Neosalanx taihuensis* (family: Salangidae, subfamily: Neosalanginae) is a small fish species native to the middle and lower reaches of the Yangtze River basin in China. The species inhabits the pelagic zone of lakes and rivers, is zooplanktivorous, and has a short generation time (∼1 y) with few individuals capable of reaching a second year of life. Over time, icefish have been introduced into lakes and reservoirs throughout China for the purpose of commercial fisheries production [26]. During 1988 and 1989, this species was introduced into Lake Erhai [27] and soon became established as one of the dominant species in the lake. Data from the Lake Erhai Protection Agency [27] documented that annual yields of the icefish increased from 530 metric tons to more than 800 metric tons over the last ten years, and now represents ∼25% of total community fish production in Lake Erhai. Unfortunately, successful establishment of icefish populations has also resulted in declines of some increasingly rare native fish species in the lake, e.g. *Cyprinus longipectoralis*, *C. pellegrini*, and *Zacco taliensis* [28].

In this study, we document life-history variations in icefish populations at three sites along the north-south axis of Lake Erhai. We exploit the natural thermal gradient present in the lake, along with the fact that each population represents recent adaptation from a single source population, as a natural experiment to explore the effect of temperature variations on the diversification of icefish life histories.

## Materials and Methods

Icefish populations were sampled monthly at three sites in Lake Erhai from June 2010 to May 2011 using a lift-net (Fig. 1). The opening gape of the net was 4 m×4.5 m with a mesh size of 3 mm. Nets were submerged horizontally, and stretched with lines by four operators on two boats at night. A lamp was set over the middle of net to assist in attracting fish. Nets were lifted after every 15 min submerged, at least 4 nets (i.e. 4*15 minutes) were lifted at each site during each monthly sampling, and a total of 150 individuals for each site and month were retained for analysis of life-history traits. Retained fish were placed over ice for later storage at −20°C in the laboratory.

Total body length (*L_T_*) and mass (*M_T_*) were measured for each individual to the nearest 0.01 mm and 0.01 g. Sex and maturation score [29] for each fish were determined via dissection under a stereomicroscope. Thirty mature females from each site were randomly selected for calculation of absolute and relative fecundity. Gonads were dissected out, and their mass (*M_G_*) and eviscerated body mass (*M_E_*) weighed to the nearest 0.1 mg. Gonad somatic index (*GSI*) was calculated using the equation: *GSI* = 100*(*M_G_M_E_* ^−1^) [30]. Oocyte diameters were measured under an optical microscope using an ocular micrometer, and the number of mature oocytes per individual (*N_O_*, defined as the total number of oocytes in the actively spawning stage) counted for estimation of absolute fecundity (*F_A_*). Relative fecundity (*F_R_*, an index measure of the fecundity of individuals relative to the body size) was calculated using the equation: *F_R_* = *N_O_M_E_* ^−1^ [31]. Condition factor (*K*) was calculated using the equation: *K* = 10^5^ *M_T_L_T_* ^−3^ [32].

Statistical differences in *L_T_*, *M_T_* and *K* among sites were evaluated using a mixed effects model repeated measures ANOVA. All *L_T_*, *M_T_* and *K* data were log-transformed to meet assumptions of normality. Thus in each model, log(*L_T_*), log(*M_T_*), and log(*K*) were dependent variables, site was a fixed variable and month of collection was a random variable. Sphericity assumption was tested using Mauchly's test and degrees of freedom were adjusted using a Greenhouse-Geisser Epsilon correction when data violated the assumption of sphericity. When significant month × site interactions were observed, one-way ANOVAs with Tukey's HSD post-hoc tests were used to evaluate among site differences in each month.

Whereas egg diameter and *F_R_* data were not normally distributed, Kruskal-Wallis tests were used to evaluate statistical differences in these variables. Significant differences among-site in these response variables were evaluated using Wilcoxon post-hoc tests. *F_A_* data were normally distributed, thus analysis of covariance (ANCOVA) was used to examine among-site differences in *F_A_* (with *L_T_* as a covariate). Significant post-hoc differences between means were determined using Tukey's HSD test. *GSI* data were normally distributed, thus one-way ANOVA with Tukey's HSD post-hoc test was used to test differences in *GSI* among-sites. Logistic regressions were used to model relationships between maturity (immature = 0, mature = 1) and total length. Lengths at which 50% of individuals were sexually mature were estimated at each site using logistic regressions (i.e, via 50% cutoff values, *L_T50_*). A log-likelihood ratio test was used to examine differences in maturity-*L_T_* relationships among the three sites [33]. Normality and homogeneity of data were determined using Shapiro's and Levene's tests. All statistical analyses were performed using SPSS v19.0 and R statistical software, version 2.14.0 and were considered significant at α<0.05.

All the procedures described in this study were approved by the ethics committee of the Institute of Hydrobiology Chinese Academy of Sciences, Hubei Province, China. Sampling permits for each location were issued by the Lake Erhai Protection Agency of China. The study did not involve any endangered or protected species.

## Results

**Figure 2:**
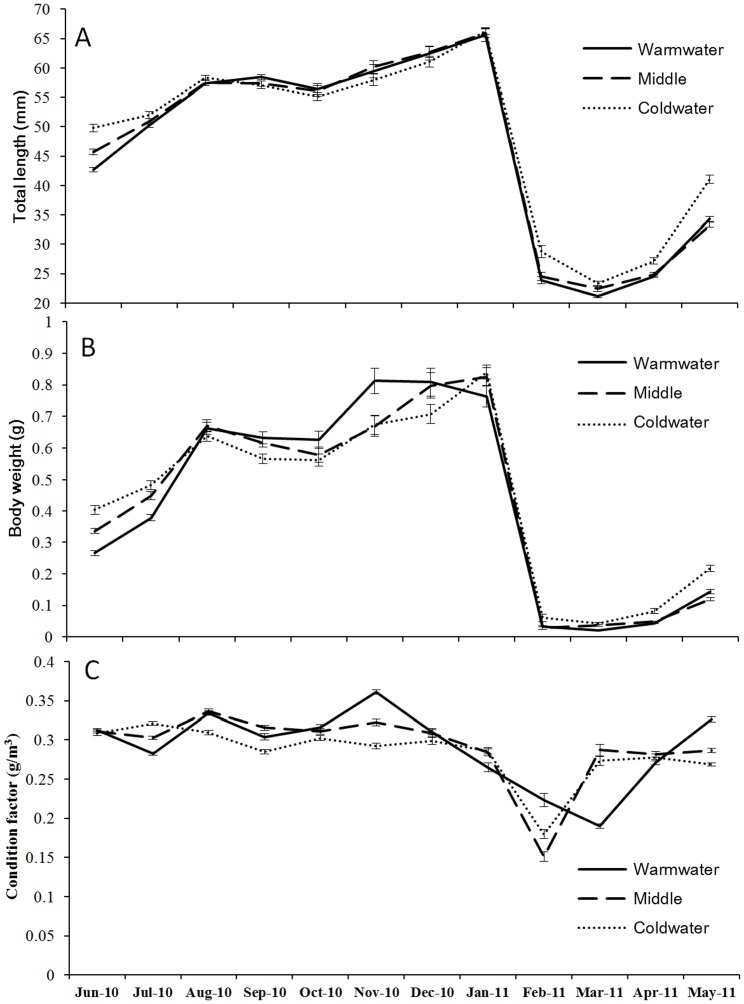
Growth performance of different icefish populations in Lake Erhai. Month by month (6/2010-5/2011) comparisons by lake section of mean (A) total length (B) body weight, and (C) condition factors of icefish in Lake.Erhai. Data are presented as mean±1 SE.

Across all months and sites, a total of 5400 individual icefish were sampled from Lake Erhai. Icefish displayed positive growth in *L_T_* and *M_T_* at all three sites over the course of the surveys (Fig. 2). At each site, *L_T_* and *M_T_* of icefish peaked in January and subsequently collapsed to the lowest values in February, thereby representing the death of the previous year's generation and birth of a new generation. There were significant differences in total length (repeated measures ANOVA F_2,5384_ = 38.84, p<0.001), body mass (repeated measures ANOVA F_2,5384_ = 23.25, p<0.001) and condition factor (repeated measures ANOVA F_2,5384_ = 5.11, p = 0.006) among all three sites.

Mean total lengths and weights (averaged across the year) were highest in the warmwater northern icefish variants followed by mid-lake icefish and finally cold water southern icefish (Fig. 2 A, B). Significant site × month interactions were evident in total length, body mass and condition factor across sites; thus one-way ANOVAs were subsequently used to evaluate differences among sites in each month separately (Table 1). Total lengths differed significantly among sites, but only during early life stages (from February to June, Table 1, Fig. 2). However, body mass differed among sites during all months with the exceptions of August and October (Table 1, Fig. 2). In contrast, mean condition factors averaged across all months were highest in warmwater northern icefish variants, followed by mid-lake, and finally coldwater southern variants. Further comparisons showed that body condition differed significantly among variants for all months evaluated (Table 1, Fig. 2).

**Table 1:** One-way ANOVAs used to test the differences in length, mass and condition factor of icefish among different sites in each month in Lake Erhai.

Month	Length			Mass			Condition factor		
	d.f.	*F*	*p*	d.f.	*F*	*p*	d.f.	*F*	*p*
1	2	0.428	0.652	2	5.875	0.003	2	16.12	<0.001
2	2	33.44	<0.001	2	15.74	<0.001	2	87.86	<0.001
3	2	3.792	0.0233	2	17.07	<0.001	2	83.19	<0.001
4	2	12.03	<0.001	2	15.07	<0.001	2	3.613	0.0278
5	2	49.59	<0.001	2	37.71	<0.001	2	68.78	<0.001
6	2	29.6	<0.001	2	20.36	<0.001	2	8.458	<0.001
7	2	2.597	0.0756	2	36.13	<0.001	2	261.8	<0.001
8	2	1.289	0.277	2	0.671	0.512	2	15.8	<0.001
9	2	1.04	0.354	2	3.233	0.04	2	17.89	<0.001
10	2	0.709	0.492	2	2.113	0.122	2	5.277	0.0054
11	2	1.406	0.246	2	10.94	<0.001	2	166.5	<0.001
12	2	2.017	0.134	2	4.952	0.007	2	4.464	0.012

**Figure 3:**
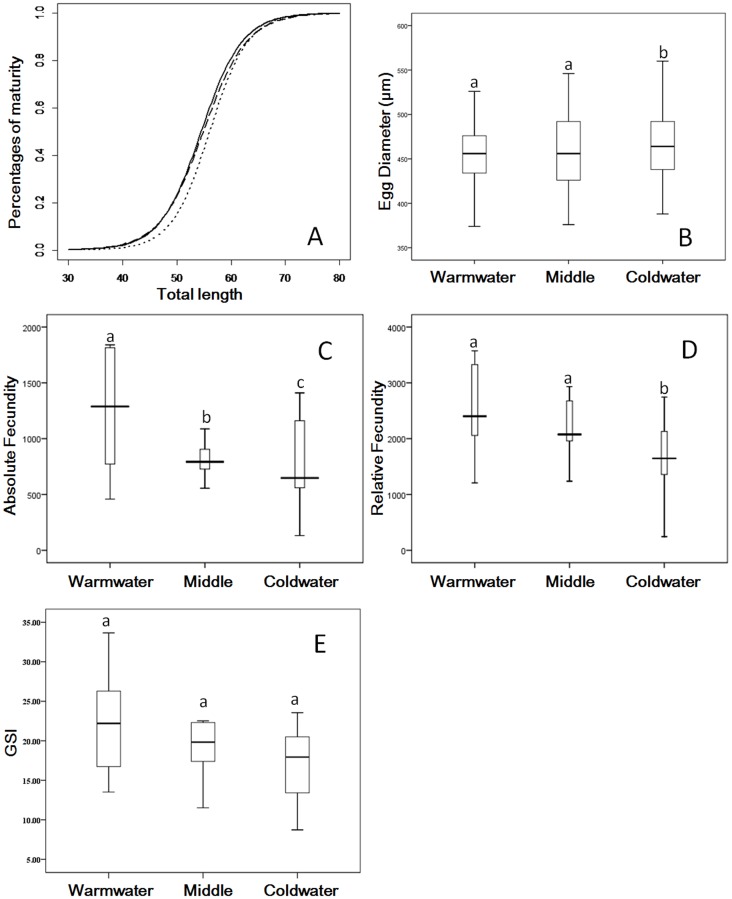
Reproductive traits of different icefish populations in Lake Erhai. (A) Predicted maturation probability for female icefish as a function of total length in the northern warmwater (solid line), mid-lake (dashed line) and southern coldwater (dotted line) sections of Lake Erhai. (B) Box plot of median egg diameter of female icefish captured from each of the three lake sections. (C) Box plot of median absolute fecundity of female icefish captured from each of the three lake sections. (D) Box plot of median relative fecundity of female icefish captured from each of the three lake sections. (E) Box plot of median gonad somatic index (GSI) of female icefish captured from each of the three lake sections. On all panels, letters denote means that do not differ significantly from one another.

Logistic models revealed that average size at maturity (*L_T50_*) increased from the warmest to coldest sites (Fig. 3). *L_T50_* values of icefish for warm, middle and cold areas of the lake were 54.5±0.8, 54.9±0.8 and 56.1±0.7 (± standard errors), respectively and log-likelihood ratio tests confirmed that differences between all three sites were significant (df = 2, p<0.001). Thus icefish variants from the warmest site matured at significantly smaller sizes relative to icefish from the coldest site.

Lake-wide, females were sexually mature and gravid during the months of October and November and showed significant among-site differences in egg diameter (Chi-squared = 23.87, df = 2, p<0.001). Coldwater icefish variants from the south produced the largest eggs, followed by the mid-lake site with intermediate-sized eggs, and finally the warmwater variants from the north with the smallest egg sizes (Fig. 3 B, Wilcoxon Test, all p's<0.01 except for the mid-lake versus northern comparison, p = 0.12). However, after accounting for body size (i.e. *L_T_* as a covariant), there were also significant differences in absolute fecundity among all three sites (ANCOVA, p<0.001, Fig. 3 C). Fecundity was highest in warmwater northern variants, followed by mid-lake icefish and finally the coldwater southern variants (Turkey's HSD Test, all p's<0.01). Relative fecundity of icefish did not differ between northern and mid-lake sites (p = 0.51), however coldwater southern icefish had significantly lower relative fecundity values than either of the other two sites (Wilcoxon Test, both p's<0.01, Fig.3 D). Similarly, GSI was highest in warmwater, followed by intermediate and finally coldwater variants, although the differences were not significant (ANOVA, df = 2, F = 2.587, p = 0.094, Fig.3 E)

## Discussion

Life-history traits are basic determinants of species and population performance [34]–[36]. Understanding how different environments shape life-history diversification is consequently foundational to the future conservation of biological diversity [37], [38]. This study documented the existence of several life-history variants of icefish across a thermal gradient in a large, montane lake in southwestern China. Coldwater icefish variants had larger sizes at maturation, slower growth rates, and reduced fecundity. Conversely, warmwater icefish variants had smaller body size at maturation, faster growth rates, and higher fecundity. Intermediate variants had life-history characteristics that were of a hybrid nature between warmwater and coldwater variants. Movement differences would be unlikely to produce the spatial variation in icefish life-histories observed as this species has a limited ability to disperse and tends to make daily movements of in the vertical rather than horizontal directions (unpublished data from another sampling). High dispersion rates would also be predicted to produce a more uniform life-history instead of spatially distinct life-history structure. We suggest that these life-history patterns be a product of rapid life-history adaptation over a period of only 25 years.

The extent to which phenotypic differences among variants observed in this study are based in genetic differences or phenotypic plasticity remains uncertain [39]. For example, average weights of Soay sheep (*Ovis aries*) declined by ∼20% due to direct temperature effects on metabolism and growth rate, rather than selection per se [40], [41]. For this reason, future research that examines genetic differences among putative icefish variants would be useful in disentangling these covarying influences. However, the degree to which all life-history parameters (egg size, fecundity, growth rate) were altered in significant and predictable ways suggests that genetic differences among variants may exist. Disentangling genetic verses environmental effects on phenotypes is a classic challenge in the study of life-history evolution [39]. For example, in the fisheries-induced-evolution literature, researchers are often relegated to quantifying reaction-norm shifts in growth rate and sexual maturation to detect evolutionary change because genetic data are lacking [42]. The patterns in growth and sexual maturation reaction norms for Lake Erhai icefish rival and exceed those commonly observed in cases of strong fisheries-induced evolution where genetic data are lacking [42]. We encourage future research that could quantify the actual genetic structure of icefish sub-stocks in Lake Erhai. Furthermore, we encourage coupling of such data with common garden experiments to evaluate how genetic and phenotypic variations are linked along with evaluating the extent to which genetic drift might be responsible for producing the life history variants [43].

Classically, a series of well-known ecological factors combine to influence the selection and diversification of life-history strategies. Below, we discuss the potential for each of several of these factors to produce the life-history patterns observed.

### 1) Spatial differences in predation risk

Predation risk can exert strong selective forces on phenotypes of prey [44]–[46]. Predators increase mortality rates of prey and thereby directly influence life-history evolution through the removal of at-risk phenotypes [47], [48]. However, predators also decrease prey density, which indirectly increases resource availability for surviving prey [48], [49]. Thus increased predation rates on juvenile age/size-classes favors the rapid evolution of fast growth and earlier sexual maturity with survivors producing increased reproductive investments in numerous smaller eggs and hatchlings [50]–[52]. Conversely, decreased predation might favor slower growth and delayed maturation with increased reproductive investments in few larger eggs less likely to be predated upon, as well as on costly sexual display behaviors [53]–[55]. However, Lake Erhai is a historically predator-poor environment. Most native fishes in this and similar ecosystems are herbivorous or omnivorous species (e.g. *Cyprinus longipectoralis*, *C. pellegrini*, *Zacco taliensis*) and lack carnivorous predator conspecifics [56], [57]. A very small number of the predacious northern snakehead (*Channa argus*) are apparently present within Lake Erhai. However, the levels are so low as to be functionally zero, and are only found in isolated areas of the littoral zone, whereas icefish exclusively occupy pelagic habitats. In fact, during a previous study of snakehead diets in Lake Erhai, none were found to have preyed upon icefish [58]. This information therefore suggests that relative predation risk did not play a role in the diversification of icefish life-history variants in Lake Erhai.

### 2) Spatial differences in ecosystem productivity

Similar to predators, variations in ecological productivity can also drive life-history diversification [44], [59], [60]. Pianka [61] long ago noted that increased productivity typically leads to r selection (i.e., high fecundity, small body size, early sexual maturity, reduced lifespan) whereas oligotrophy often yields K selection (i.e., low fecundity, larger body size, later sexual maturity, increased lifespans). While the field of life-history theory has greatly advanced, the basic tenets of r/K selection theory remain relevant as a heuristic tool for understanding basic life-history shifts. Thus, if spatial patterns in productivity were driving diversification of icefish life histories in Lake Erhai, the faster-growing, shorter-lived icefish variant would have been predicted to have originated from the most productive environment. Yet, when data on phosphorus, chlorophyll α concentrations, and zooplankton abundances were examined (unpublished data), an opposite pattern was evident. The more “r-selected” warmwater northern icefish population in fact originated from the least productive sections of the lake, and the more “K-selected” coldwater southern population from the most productive section of the lake. Thus, it remains highly unlikely that life-history diversification of icefish in Lake Erhai is related to spatial patterns in productivity. In fact, because the ecological productivity patterns are operating counter to theory, it remains plausible that life-history variants would have potentially been even more exaggerated had there been lake-wide neutrality in productivity.

### 3) Fisheries-induced evolution

Under heavy exploitation pressure, fish populations are known to evolve in characteristic ways [62]–[64]. For example, body size within populations often declines with increasing fishing activities, since fisheries typically target the largest individuals [62]. Under such scenarios, parental effects can subsequently ensue whereby the smallest, slowest-growing phenotypes and genotypes are left behind to contribute disproportionately to the population over time [65]. Theory also predicts that a general reduction in size that cascades to a genetic level would likely be accompanied by shifts towards earlier sexual maturation schedules [42], [66]. There were clear differences in icefish size and maturity across Lake Erhai as the northern warmwater variants were significantly larger and had a higher percentage of mature fish at smaller sizes relative to other populations. Thus smaller fish were not necessarily more mature as a fisheries-induced evolution hypothesis would have predicted. Furthermore, the commercial fishery pressure on Lake Erhai is roughly equivalent across the lake as fishers frequent multiple sections of lake with varying effort. In light of the conflicting life-history response and relatively equal fishing pressure lake-wide, it seems unlikely that fisheries-induced evolution is playing a role in the diversification of life-history strategies in icefish in Lake Erhai.

### 4) Spatial differences in thermal environments

Temperature frequently varies substantially within aquatic ecosystems [16], [17]. For example, thermal and chemical stratification of water in lake ecosystems frequently produces a cool, oxygen-rich layer of water (i.e., the thermocline) that serves as thermal refugia for diverse cool and coldwater fish species [67], [68]. Considerable work has focused on the seasonal structuring of these habitats and how anthropogenic drivers (e.g., phosphorus enrichment) may interact with temperature to limit the availability of these habitats [68]-[70]. However, as seen in Lake Erhai and other ecosystems, temperature can also vary significantly in the horizontal dimension across the spatial area of a single large lake. Yet horizontal variations in lake temperature are not nearly as well-studied in lakes as vertical variations in temperature, even though these variations can produce analogous limits on habitat availability.

Life-history theory predicts that a significant thermal gradient in a large lake environment (given sufficient selection strength and isolation) could, over time, elicit the formation of predictable and divergent life-history strategies [34], [59], [71]. For example, in cool- and cold-water fish species, coldwater environments typically produce large-bodied life-history variants with slower somatic growth rates [72]–[74]. Increased body size is presumed to be a product of reduced rates of natural mortality [11], perhaps due to reduced oxidative stress stemming from slower growth rates [75]. Thus while growth may be slower, over time, individuals can actually achieve larger body sizes (i.e., the temperature-size rule) [6]. Individuals in colder environments also delay maturation, generating reduced fecundities at a given age [76]. The reduction in fecundity in these variants is “balanced out” by increased lifespan and egg size [77]–[80]. The life-history variants of icefish found across Lake Erhai aligns with these life-history theory predictions. Combined, with a lack of evidence in support of the other potential explanations, we suggest that icefish life histories have indeed diversified rapidly across Lake Erhai in response to the horizontal water-temperature gradient.

A variety of examples of rapid evolution exist in response to thermal and other ecological changes against which these results might be compared [81], [82]. In *Drosophilia subobscura* in the southern Palearctic, chromosomal diversity was estimated to have shifted by 18.3% in only 16 years as a result of rapid global warming in this region [81]. Furthermore, populations of a frog (*Rana sylvatica*) underwent rapid microgeographic evolution in thermal tolerance in only 36 y following the construction of beaver ponds and corresponding alteration of thermal water regimes [83]. When taken to extremes, evolutionary shifts (that align with theoretical predictions of selection effects on phenotypes) can even be achieved within a single generation [84].

### 5) Adaptive evolution or genetic drift

Ultimately, it also remains unclear whether phenotypic differences and putative genetically-based life histories were a product of true adaptive evolution or genetic drift (i.e., a change in the allelic frequency of a population due to random sampling) or a combination of both. Both processes are well-known to lead to genetic differentiation in a diverse number of taxa and situations [85]–[87]. Certainly at low population levels, the risk for genetic drift to play a larger role in the evolution of icefish sub-stock genetics increases, i.e., due founder effects [88], [89]. However, the convergence of life-history patterns upon phenotypes commonly reported within the literature at macroecological scales due presumably to temperature as a selective pressure is compelling [90]–[92]. Indeed, the icefish example aligns well with the hypothesis of Reznick and Ghlambor [93] that common examples of rapid evolution typically involve both a heterogeneous environment that encourages selection along with a short-term opportunity for explosive population growth. Without additional genetic and common garden experimental work, however, many of these questions will remain unanswered.

Species introductions can also be a major driver of rapid evolution and diversification of phenotypes [94]. A lack of appreciation for rapid evolution is in fact viewed as a primary impediment to improved predictions of successful establishment and niche models of invasive species at large spatial scales [94]–[96]. Concordant with a review on this topic [94], we report relatively rapid rates of adaptation for an introduced species. This is unsurprising given the rapid generation time of icefish (∼1 y), thus representing roughly 25 generations over which diversification could have potentially occurred in Lake Erhai. This study therefore represents yet another example of the potential for introduced species to adapt to novel environments, even when conditions (e.g. thermal habitat) are exceptionally heterogeneous. It remains uncertain whether the original icefish stock introduced into Lake Erhai most closely resembled the warmwater, coldwater or hybrid variant in its life-history. However within its native range, the species is commonly encountered in warmwater lotic environments (e.g., within the main channel of the lower Yangtze River). Thus, the species is likely adapted to a high degree of habitat heterogeneity such as commonly occurs in large floodplain rivers [97], [98]. Contrasting with this adaptive potential are the life-histories of the native lentic fishes of Lake Erhai, some of which have average generation times >40 y [56], and thus a much lower potential to adapt to environmental change.

Previous research on life-history evolution of aquatic organisms in response to thermal-scaling laws originates primarily from macroecological studies, e.g., across species ranges or continents [9], [99], [100]. For example, in the northern hemisphere, many organisms at northern latitudes have larger body sizes, increased longevity and delayed sexual maturation and these traits typically scale with declining latitude [100], [101]. These patterns are rarely examined at microgeographic scales in poikilothermic animals (but see [14], [102]–[104]. However, it has long been known that life histories of myriad terrestrial plant and animal species change predictably along elevational clines [105], [106]. Some examples also exist within marine fisheries, even though the spatial scales of these studies are primarily macroecological as well [107]–[109]. Our data for Lake Erhai icefish suggest that microgeographic (horizontal) variations in water temperatures can similarly operate on key elements of fish biology and that these variations should be considered when working in large ecosystems where temperature varies significantly in space.

These findings have bearing on the conservation management of ectothermic animals in large ecosystems. For example, fisheries management in large lakes is typically conducted on lake-wide scales. Even in extremely large ecosystems (e.g., the Laurentian Great Lakes), fisheries regulations can vary little system-wide. However, these data show that, within large lake environments, fish life histories can be quite different depending on the thermal characteristics of the lake section. These dynamics foreshadow new challenges for fisheries managers that require additional thought and research. For a variety of reasons, it can be unpopular to manage different lake sections under different fishing regulations. Furthermore, limited budgets and staffing issues in water-rich areas preclude collection of intense system-specific data on fish populations. Nonetheless, when life-history variants exist under a single management system, the potential for poor management of one of the variants will be present (typically the slower-growing, long-lived variants under liberal harvest regulations) [108], [110]. Future theoretical, population-dynamic, and human-dimensions research on such scenarios would benefit managers challenged with spatially complex fisheries exhibiting multiple life-history variants.

Enhanced reproduction as a consequence of reduced body size may also be an important pattern for understanding biological response to climate change. Currently, a large amount of research is being conducted towards understanding species geographic-range shifts under various warming scenarios [111], [112]. However, as this study illustrates, increased temperatures can also change species life histories at microgeographic scales (even within a single large ecosystem). Concurrent with this study, a recent study in ocean environments highlighted the probability of species body-size shrinkage in response to warming temperatures [113]. All species possess a capacity to evolve under changing environmental conditions, yet persistence depends on a rate of adaptation that out-strips that of environmental change [23]. In a species like icefish, adaptation to thermal changes is realistic given the rate at which selection (or plastic adaptation) can apparently occur. However, in more long-lived species (e.g., native Cyprinidae in coldwater montane lakes of China), adaptation may not be plausible. Understanding the limits of life-history adaptation to climate change will therefore be central to the prioritization of various conservation management efforts and in projecting climate change impacts on diverse species and populations.
